# First human whole-body biodistribution and dosimetry analysis of [^18^F]LW223, a novel TSPO PET radiotracer

**DOI:** 10.1007/s00259-025-07722-0

**Published:** 2026-01-10

**Authors:** Phyo H. Khaing, Mark G. MacAskill, Jianfei Xiao, Shichao Liu, Zhuqin Gu, Xiaohiu Sun, Tao Xu, Norman Koglin, Andrew W. Stephens, David E. Newby, Yihui Guan, Holly McErlain, Andrew Sutherland, Gilles D. Tamagnan, Fang Xie, Adriana Alexandre S. Tavares

**Affiliations:** 1 Institute for Neuroscience and Cardiovascular Research, University of Edinburgh, Edinburgh, UK; 2 Edinburgh Imaging, University of Edinburgh, Edinburgh, UK; 3 Department of Nuclear Medicine & PET Center, Huashan Hospital, Fudan University, Shanghai, China; 4 XingImaging LLC, New Haven, CT USA; 5 Life Molecular Imaging GmbH, Berlin, Germany; 6 School of Chemistry, University of Glasgow, Glasgow, UK; 7 Institute for Neuroscience and Cardiovascular Research, Queen’s Medical Research Institute, 47 Little France Crescent, Edinburgh, EH16 4TJ UK

**Keywords:** TSPO, [^18^F]LW223, Dosimetry, Whole-body, Human, Biodistribution

## Abstract

**Purpose:**

The 18 kDa translocator protein (TSPO) has been a central molecular target for imaging inflammation in the preclinical and clinical research settings across a plethora of applications, including neuroinflammation, cardiovascular inflammation and cancer. Recently, we reported the development of [^18^F]LW223 as a third-generation TSPO positron emission tomography (PET) radiotracer with binding to human TSPO independent of the rs6971 genetic polymorphism. This study reports the first whole-body human analysis, including biodistribution and dosimetry calculations, following intravenous administration of [^18^F]LW223.

**Methods:**

Whole-body PET images were acquired over 250 min after intravenous bolus injection of 184.3 ± 20.2 MBq of [^18^F]LW223 in healthy adult human volunteers. Volumes of interest (VOIs) in different source organs were manually delineated by three independent observers, then time-activity curves were generated for residency times calculations for subsequent quantification of radiation equivalent and effective doses using OLINDA/EXM 2.2 software.

**Results:**

The radiotracer biodistribution in humans recapitulated known TSPO expression in various tissues. The main elimination route was found to be hepatobiliary, and the critical organ was the intestine. The cumulated radioactivity excreted by the kidneys was < 10% over the measurement period and no bone uptake suggestive of in vivo defluorination was observed in any of the study subjects. The effective dose ranged between 11.8 ± 0.9 and 12.5 ± 0.9 µSv/MBq. Inter-observer VOI variability had no impact on estimated organ and whole-body effective doses.

**Conclusion:**

[^18^F]LW223 is predominantly excreted by the hepatobiliary route with no evidence of in vivo defluorination but demonstrates marked uptake into tissues with known TSPO expression. It complies with radiation limits and guidelines recommended by regulatory authorities and is in line with previously reported [^18^F]-labelled radiotracers, such as [^18^F]fluorodeoxyglucose. [^18^F]LW223 is suitable for translation into human clinical studies.

**Supplementary Information:**

The online version contains supplementary material available at 10.1007/s00259-025-07722-0.

## Introduction

The quest for improved 18 kDa translocator protein (TSPO) positron emission tomography (PET) radiotracers has been a long and tortuous journey. Since the discovery of TSPO ligands around 40 years ago, various compound libraries have been developed for imaging TSPO [1, 2]. The seminal ligand, [^11^C]PK11195, has several shortfalls precluding widespread clinical use. These include high non-specific binding and labelling with a short-lived radioisotope [3]. Through a major community effort over decades, several second-generation radiotracers have been developed for imaging TSPO with PET. Second-generation TSPO ligands were successful at addressing some of the shortfalls, including improved non-specific binding characteristics (e.g. [^11^C]PBR28 [4, 5]) and labelling with longer-lived radioisotopes (e.g. [^18^F]DPA714) [6, 7]. However, these second-generation TSPO ligands suffered from the major drawback that human TSPO binding was dependent of the rs6917 genetic polymorphism [8]. In practice, this meant that a substantial percentage of the human population could not be imaged with second-generation TSPO PET ligands, as they displayed either no binding (Low Affinity Binders, LABs) or two-site binding (Mix Affinity Binders, MABs) profiles [9].

In the past decade, third-generation TSPO ligands have been explored with the main focus of achieving human TSPO binding that was independent of the rs6971 genetic polymorphism, had good physicochemical properties and could be labelled with fluorine-18 for easier widespread clinical adoption. Among these third-generation ligands, [^18^F]LW223 was the first to show binding to human TSPO independent of the rs6917 genetic polymorphism [10]. To our knowledge, this novel radiotracer has the lowest non-specific binding [11] and highest sensitivity for detection of TSPO changes [12] among TSPO PET radiotracers. It also has low radiometabolism across species [10, 11, 13] and can be easily radiolabelled with fluorine-18. Proof-of-concept human brain studies with [^18^F]LW223 also showed this radiotracer was brain penetrant with suitable reversible kinetics in vivo [13].

Although previous studies have shown the potential of [^18^F]LW223 as a superior TSPO PET radiotracer, whole-body human biodistribution and dosimetry analysis for this radiotracer is lacking. Dosimetry estimates are important to establish the safety of the radiotracer administration into subjects and to ensure the subjects’ radiation exposure levels are within acceptable limits. Typically, these are conducted using Volumes of Interest (VOIs) drawn by a single imaging scientist, however, such methodology may be prone to inter-observer errors [14–16], therefore multi-observer analysis can be valuable to derive the most accurate dosimetry estimates and safety confidence intervals. This study presents the first human whole-body biodistribution and dosimetry analysis with [^18^F]LW223. This was done using VOIs drawn by three independent imaging experts, in order to derive accurate dosimetry estimates.

## Materials and methods

### Radiochemistry

LW223 precursor, (*R*)−3-chloromethyl-(*N*-*sec*-butyl)-*N*-methyl-4-phenylquinoline-2-carboxamide was prepared in eight steps from aniline and diethyloxalpropionate as previously described [10]. The general approach involved initial synthesis of the 4-hydroxyquinoline core, a Suzuki-Miyaura cross-coupling reaction to incorporate the phenyl moiety, side-chain amide construction and a Wohl-Ziegler halogenation as the key steps. Radiolabeling and preparation of [^18^F]LW223 was described in detail previously [10]. In brief, radiofluorination was performed using [^18^F]fluoride in the presence of K222, using a PET-MF-2 V-IT-I synthesizer. Purification was done via semipreparative HPLC using a C18 Synergi Hydro-RP 80 Å, 150 × 10 mm, 4 μm column. Formulation of [^18^F]LW223 was achieved using 10% ethanol in saline. Quality control showed radiochemical purity > 99% for all the productions. The average decay-corrected radiochemical yield was 26.1% ± 5.9% (*n* = 5) in 60 min.

### Human subjects

Study protocols were reviewed and approved by the Institutional Ethical Reviewing Board of the Huashan Hospital. All subjects gave their written informed consent before participation in this study.

Six healthy adult male human volunteers (based on medical history, physical examination and blood biochemistry), with mean age of 33.2 ± 2.6 years (range 30–36 years old), body weight of 67.7 ± 2.7 kg (range 63–70 kg) and height of 173.7 ± 4.5 cm (range 165–178 cm), were enrolled in this study. None of them had known clinical history that could have affected the biodistribution or elimination of [^18^F]LW223.

### Whole-body PET acquisition and reconstruction

Whole-body PET images were acquired on a Siemens Biograph mCT Flow 128 Edge scanner. Healthy controls received a bolus intravenous administration of [^18^F]LW223 (184.3 ± 20.2 MBq [range, 153.7–210.6 MBq]), immediately followed by a series of whole-body PET images consisting of 13 contiguous bed positions. Images were acquired over approximately 250 min in 2 scanning sessions, with subjects being allowed out of the camera for 20 min between sessions. The first session included 9 whole-body passes (2 × 15 s, 2 × 30 s, 2 × 60 s, 2 × 120 s, and 1 × 240 s per bed position), and the last session included 1 whole-body pass (1 × 480 s per bed position). A CT scan was acquired at the end of each PET imaging session. The following parameters were used for CT acquisition and reconstruction: 100 kVp, 166 mAs, i30f filter, pitch of 0.6 and Filtered-Back Projection (FBP). PET data were corrected for photon attenuation, scatter and random and reconstructed using OSEM (4 iterations, 24 subsets) with Gaussian filter with a kernel of 5 mm and a matrix size of 400 × 400. PET data were also reconstructed using FBP with a Butterworth filter with order 2 and kernel of 20 with the same matrix size as OSEM reconstructions.

Urine was collected for up to 5 h post radiotracer injection to measure the fraction of activity voided by the renal system. Activity not excreted in urine was assumed to be eliminated in faeces. Excreted urine volume was recorded for each subject and samples (1 mL each) were measured by gamma counting (PerkinElmer 2470, Perkin Elmer, USA). Measured counts per minute (CPM) were decay corrected to time of radiotracer injection. The percentage injected dose (%ID) in urine was estimated using a CPM to disintegration per minute (DPM) factor of 0.46 and the total injected dose administered to that subject.

### Whole-body PET analysis

Reconstructed whole-body PET and CT images were imported into PMOD AudiTrail 4.5 (PMOD Technologies, Switzerland). For each subject, whole-body PET images from the second imaging session were merged with the first imaging session to produce dynamic PET datasets with 10 frames. Coarse rigid motion correction was applied to all frames to ensure VOIs propagate successfully across both imaging sessions. When this was not possible due to positional changes between session 1 and 2, VOIs were dynamically adjusted for each frame to ensure they captured all source organs successfully, while keeping their shapes and sizes constant. Source organs were defined as organs with higher radioactivity concentration than background. The following organs were identified as source organs: brain, salivary glands, tongue, thyroid, heart, lungs, gallbladder, liver, spleen, kidneys, urinary bladder, intestine and red marrow. With exception of the red marrow VOI, all VOIs were manually drawn on the PET images averaged from frame 1 to 5 (to define brain) or 1 to 9 (to define all organs except brain). Red marrow VOIs were defined using CT imaging and a Hounsfield Unit (HU) range of 115 to 300 HU, in agreement with previous publications [17]. Red marrow was segmented, as it was a clear source organ with increased [^18^F]LW223 uptake and this agrees with known TSPO expression in that tissue. A whole-body VOI was drawn around the subject body and was used for quantification of whole-body remainder activity as whole-body activity minus source organs activity. VOIs were drawn manually by three observers/imaging experts independently of each other using the same set of whole-body PET/CT images.

Non–decay-corrected time–activity curves were generated for each source organs, expressed as percentage injected dose (%ID). After the last measured time point, elimination was assumed to be through physical decay only. The residence time τ, defined as the ratio of accumulated activity in the source organ (Ā) and injected activity (A_0_); τ=Ā/A_0_, was calculated as the area under the curve of the tissue (TAC), from time zero to infinity using the trapezoidal method. Radiation equivalent dose (Eq. 1) and effective dose (weighted average using ICRP-103 tissue-weighting factors on [18]) were estimated with the OLINDA/EXM 2.2 software package (Hermes Medical Solution, Sweden) [19] using the calculated τ. The tissue weighting factor (*w*_*T*_) for the remainder tissues (0.12) applies to the arithmetic mean dose of the 13 organs and tissues for each sex listed in ICRP-103.

$$\:{H}_{T}=\sum\:_{R}{w}_{R}{D}_{T,R}$$

As per ICRP-103 definitions, *H*_*T*_ refers to equivalent dose, *D*_*T, R*_ is the mean absorbed dose from radiation *R* in a tissue or organ *T*, and *w*_*R*_ is the radiation weighting factor. For photons and electrons, the recommended *w*_*R*_ is 1. Since *w*_*R*_ is dimensionless, the unit for the equivalent dose is the same as for absorbed dose, J·kg^− 1^, and its special name is sievert (Sv).

### Data and statistical analysis

GraphPad Prism, version 9 (GraphPad Software Inc., USA), was used for data fitting, statistical analysis, and production of graphs. Two-way ANOVA with post hoc Šidák correction was used in this study for comparison between observers, as indicated within the relevant figure legends, with a *p* value of less than 0.05 considered statistically significant. All error bars represent the SEM, unless otherwise indicated in the figure or table legends.

## Results

### Whole-body biodistribution and dosimetry

**Fig. 1:**
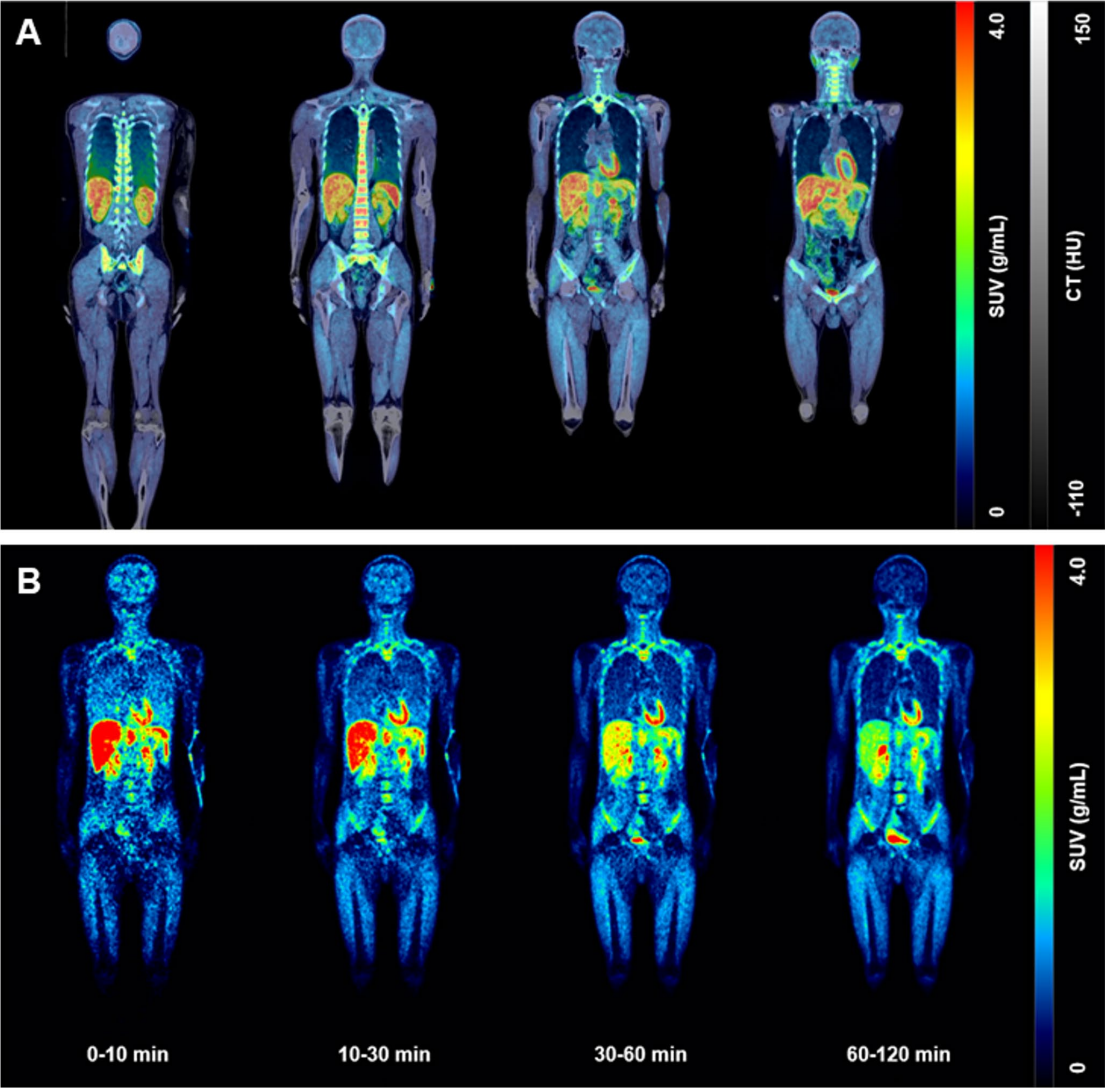
Representative decay-corrected human whole-body PET images following intravenous administration of [^18^F]LW223. (**A**) Average whole-body human SUV PET images (0–60 min) across multiple slices and overlayed onto CT images. (**B**) Whole-body human SUV PET images averaged at different time point intervals post-injection

[^18^F]LW223 human whole-body distribution revealed several source organs (Fig. 1A) following early radiotracer distribution in blood pool with elimination of the radioactivity mainly via the hepatobiliary system (Fig. 1B).

**Fig. 2:**
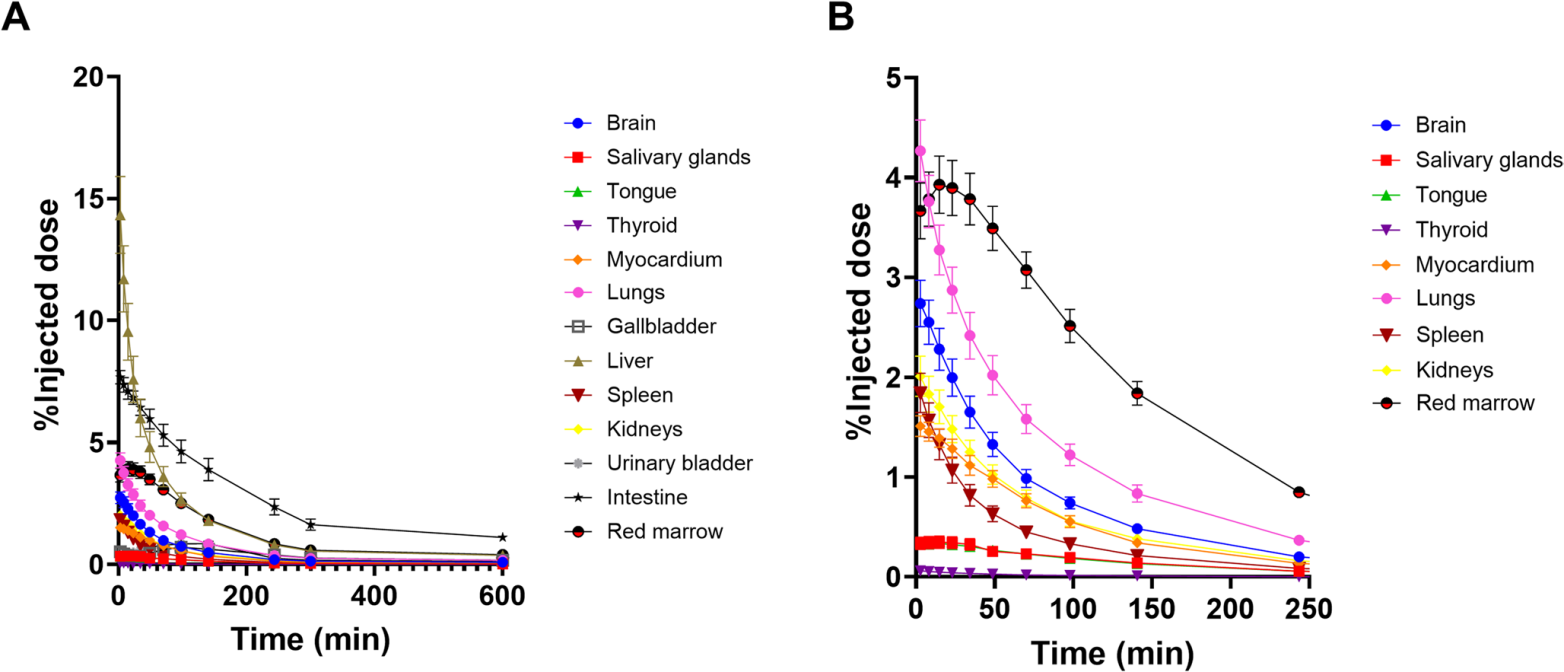
Human adult male non-decay corrected time-activity curves in different source organs (**A**) and different target-expressing tissues (**B**) following intravenous bolus injection of [^18^F]LW223. Data presented as mean ± SEM, *n* = 6

**Fig. 3:**
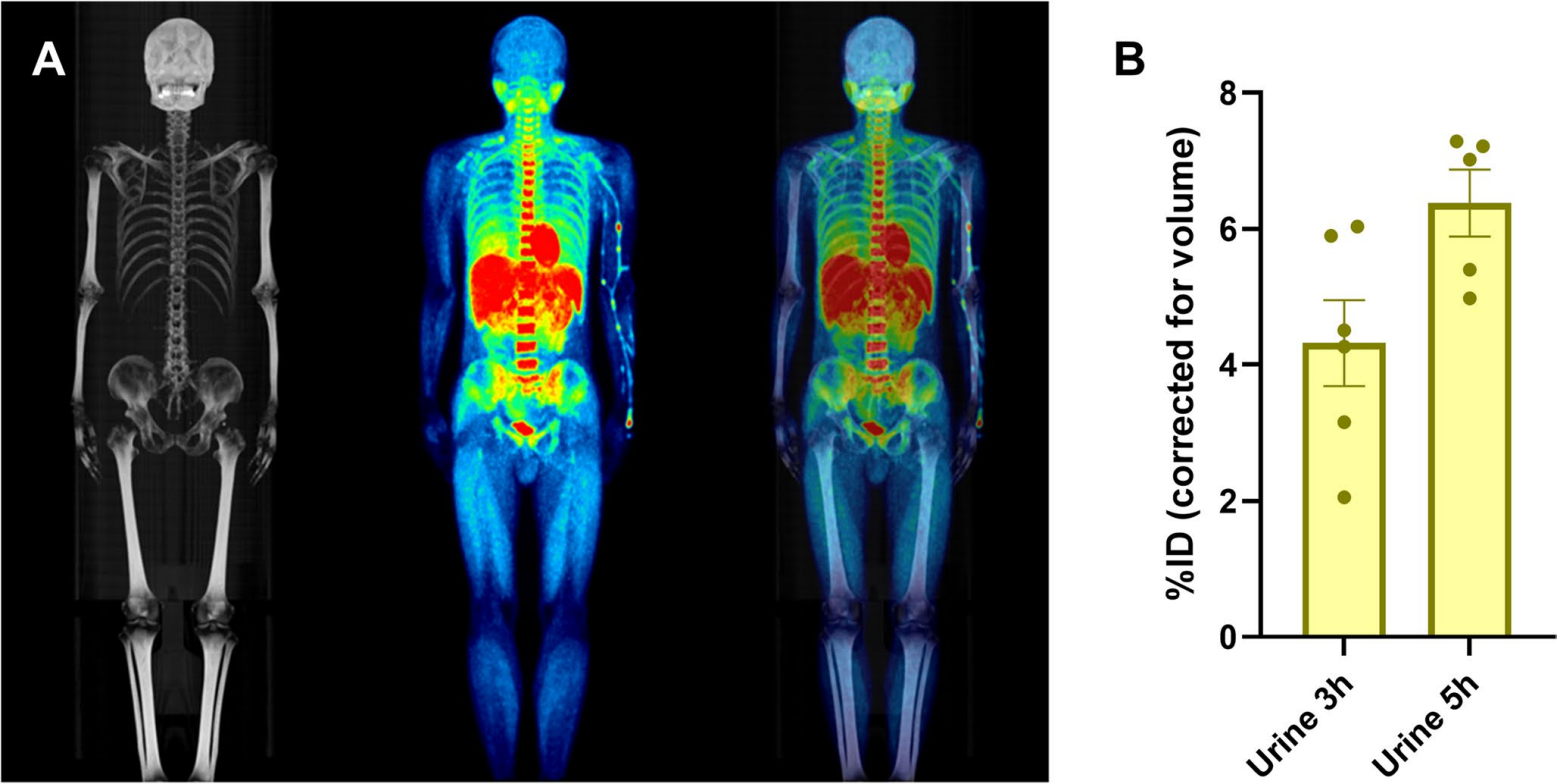
[^18^F]LW223 in vivo distribution and urinary excretion. (**A**) Maximum intensity projection (MIP) CT image, decay-corrected whole-body PET maximum intensity projection SUV average image (0–60 min) of [^18^F]LW223 distribution in an adult human male and overlay of CT and PET MIPs. (**B**) Percentage injected dose (%ID) corrected for total urine volume void up to 5 h post-radiotracer injection. Data presented as mean ± SEM, *n* = 5–6

The τ values were highest for the liver and intestine (Table 1), with a peak %ID in the liver of 14.3 ± 3.8% at 5 min post radiotracer injection (Fig. 2A). Peak %ID in other organs were much lower, with 7.7 ± 0.7% in the intestine, 4.2 ± 0.7% in the lungs, 3.9 ± 0.7% in the red marrow, 2.7 ± 0.6% in the brain, 2.0 ± 0.5% in the kidneys, 1.8 ± 0.5% in the spleen, 1.5 ± 0.2% in the heart, and all other organs below 1.0% (Fig. 2A-B). There was no observable evidence of defluorination (Fig. 3A) and less than 10% cumulative decay-corrected %ID was measured in the urine by 5 h post-radiotracer injection (Fig. 3B).

**Table 1:** [^18^F]LW223 residence times (τ) for all human source organs. Data presented as mean ± SD, *n* = 6

Organ	Residence times (τ)
Brain	0.0353 ± 0.0056
Gallbladder Contents	0.0230 ± 0.0253
Small Intestine	0.2008 ± 0.0413
Heart Wall	0.0225 ± 0.0042
Kidneys	0.0282 ± 0.0057
Liver	0.1372 ± 0.0358
Lungs	0.0577 ± 0.0106
Salivary Glands	0.0062 ± 0.0024
Red Marrow	0.0970 ± 0.0120
Spleen	0.0172 ± 0.0041
Thyroid	0.0008 ± 0.0003
Urinary Bladder Contents	0.0283 ± 0.0134
Total body	1.6148 ± 0.0813

Table 2 lists the average equivalent doses and the effective dose for [^18^F]LW223 in adult male humans. The mean effective dose for a human subject was 12.3 ± 0.9 µSv/MBq, with the small intestine receiving the highest equivalent dose of 62.3 µSv/MBq.

**Table 2:** Radiation equivalent dose estimates and whole-body effective doses for [^18^F]LW223 in the adult human male. Data presented as mean ± SD, *n* = 6

Organ	Estimated dose (µSv/MBq)
Adrenals	15.62 ± 1.20
Brain	7.82 ± 0.87
Esophagus	10.67 ± 0.32
Eyes	7.51 ± 0.31
Gallbladder Wall	52.72 ± 42.81
Left colon	15.50 ± 0.98
Small Intestine	62.33 ± 10.69
Stomach Wall	11.85 ± 0.33
Right colon	14.00 ± 0.86
Rectum	12.70 ± 0.62
Heart Wall	18.95 ± 2.17
Kidneys	24.38 ± 3.64
Liver	23.48 ± 5.23
Lungs	13.82 ± 1.53
Pancreas	15.37 ± 0.82
Prostate	11.65 ± 0.46
Salivary Glands	16.48 ± 4.56
Red Marrow	15.43 ± 0.80
Osteogenic Cells	12.90 ± 0.55
Spleen	27.00 ± 4.76
Testes	8.34 ± 0.33
Thymus	9.93 ± 0.32
Thyroid	11.83 ± 2.06
Urinary Bladder Wall	23.53 ± 6.30
Total Body effective dose (as per ICRP-103)	**12.28 ± 0.91**

## Impact of multi-observer VOI definition on dosimetry outcomes

**Fig. 4:**
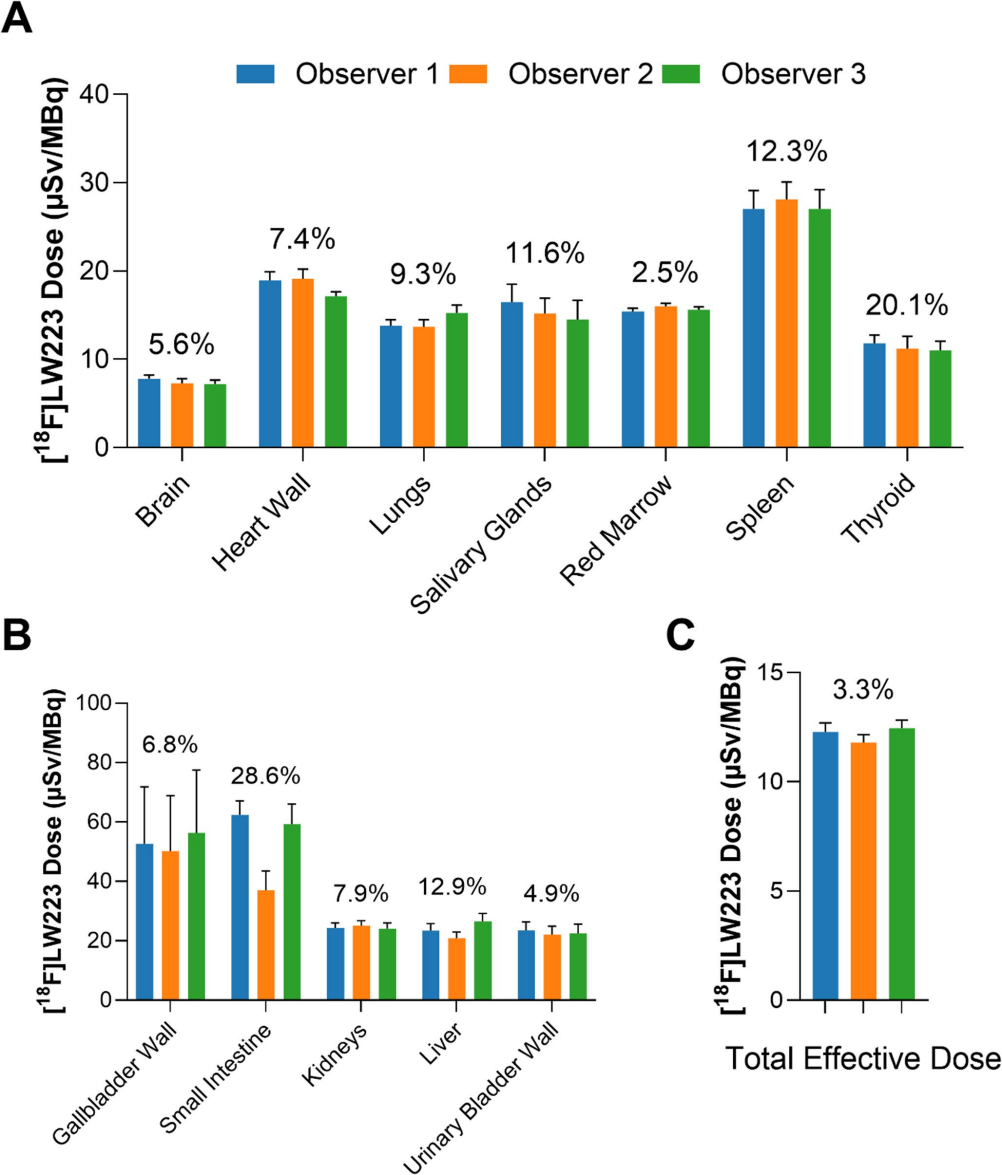
Inter-observer reliability analysis of [^18^F]LW223 dosimetry scans. (**A**) Radiation doses measured in TSPO expressing organs across three observers. The values shown in all panels are the % coefficient of variance between the observers. No significant differences were evident between observers (*p* = 0.733, two-way ANOVA). (**B**) Radiation doses measured in [^18^F]LW223 elimination organs. No significant differences were evident between observers (*p* = 0.4570, two-way ANOVA). (**C**) Total body effective dose, with again no difference evident between the three observers (*p* = 0.4660, one-way ANOVA). The results are shown as the mean ± SEM and *n* = 6 for all panels

The organ volumes manually segmented by three independent observers had an average % coefficient of variance (%CV) of 12.9% (range 3.1–43.9%, Supplementary Table 1). In agreement with organ volume data, measured τ values had an average %CV of 10.0% (range 3.0–33.3%, Supplementary Table 2). Overall, the impact of multi-observer analysis on the estimated radiation doses was low, and there were no statistically significant differences (Fig. 4), although the relatively small number of observers (*n* = 3 in this study) may limited study power. The variance seen across the TSPO expressing organs (Fig. 4A) was generally lower than the variance seen across the elimination organs (Fig. 4B) between the three observers. The estimated whole-body effective doses ranged between 11.8 ± 0.9 and 12.5 ± 0.9 µSv/MBq (mean ± SD) across the observers (Fig. 4C).

## Discussion

These are the first in vivo human whole-body PET studies conducted to evaluate [^18^F]LW223 biodistribution and elimination properties as well as dosimetry estimates in healthy human volunteers. [^18^F]LW223 distribution in the whole body was consistent with known TSPO densities [20] and with our prior observations in total-body PET studies in rodents [10]. [^18^F]LW223 displayed suitable dosimetry profile for translation into clinical use.

Whole-body PET imaging studies following intravenous injection of [^18^F]LW223 in healthy human volunteers showed that this radiotracer main elimination route was hepatobiliary. The determined mean effective dose of ~ 0.012 mSv/MBq suggests only low to modest radiation exposure associated with [^18^F]LW223 imaging in humans and allows for multiple scans to be performed in the same research subject per year. Furthermore, the determined effective dose is similar to that of other ^18^F-labelled radiotracers currently used in human studies. For example, the whole body effective dose determined following intravenous injection of [^18^F]fluorodeoxyglucose is around 0.029 mSv/MBq [21].

Compared with other previously developed TSPO PET radiotracers, [^18^F]LW223 biodistribution in source organs was comparable to [^11^C]PK11195 [22], [^18^F]PBR06 [23], [^18^F]PBR111 [24], [^18^F]FEPPA [25] and [^18^F]FEDAA1106 [26]. With regards to critical organ analysis of ^18^F-labelled TSPO ligands, the gallbladder wall received greater radiation dose from studies conducted with [^18^F]PBR06 (367.0 µSv/MBq) versus [^18^F]PBR111 (17.25 µSv/MBq), [^18^F]FEPPA (16.1 µSv/MBq), [^18^F]FEDAA1106 (27 µSv/MBq) and [^18^F]LW223 (52.72 µSv/MBq). The small intestine received more radiation exposure with [^18^F]FEDAA1106 (60 µSv/MBq), [^18^F]PBR111 (40.68 µSv/MBq), and [^18^F]LW223 (62.3 µSv/MBq) versus [^18^F]FEPPA (11.8 µSv/MBq) and [^18^F]PBR06 (13.3 µSv/MBq). Undoubtably these changes in source organ exposures are dependent on inter-subject physiological differences as well as physicochemical nature of the ligands. Overall, human radiation exposure with [^18^F]LW223 (total effective dose of ~ 12 µSv/MBq) is of the same order as [^18^F]PBR111 (16.17 µSv/MBq), [^18^F]PBR06 (18.5 µSv/MBq) and [^18^F]DPA714 (17.2µSv/MBq) [27], and lower than [^18^F]FEPPA (21.0 µSv/MBq) and [^18^F]FEDAA1106 (36 µSv/MBq).

We observed no bone uptake suggesting no major radiometabolism and defluorination of [^18^F]LW223 in humans. This is contrary to previously published studies of [^18^F]LW223 whole-body distribution in non-human primates, where potential defluorination was suggested as root cause of bone uptake [28]. The discrepancy of the observations may stem from anatomical mis-interpretation of red marrow uptake, which is expected for TSPO radiotracers [20], as bone uptake. In this regard, delineating VOIs in bones without thresholding by HU may introduce bias in the separation of cortical bone uptake versus marrow uptake. The scan length for the non-human primate study (to mid-thigh) may also have contributed to previous results mis-interpretation, as most of the red marrow is present in axial bones rather than appendicular bones [29]. It is also possible that species differences contribute to the observed discrepancy between our human whole-body data and the non-human primate study previously published. In our human study, the whole body (from tip of head to toes) was included, revealing no bone uptake by appendicular bones, including long bones in the legs. Cortical bone VOI analysis was not carried out as it was not an identifiable source organ through visual inspection of the PET images. It is possible that low-level defluorination could be present, although urine analysis suggest that would be a cumulative maximum of < 10% at ~ 5 h post-injection. Our previous studies in mice and rats indicate low radiometabolism of [^18^F]LW223 and no observable bone uptake suggestive of defluorination [10, 11].

## Conclusion

Data reported here demonstrated that [^18^F]LW223 increased uptake in different tissues, including brain, heart, kidneys, spleen and red marrow, is consistent with binding to intended target, suggesting it could be a suitable PET radiotracer for in vivo imaging of TSPO in humans. Estimated mean effective dose suggests that an acceptably low radiation exposure is associated with [^18^F]LW223 imaging in human subjects and it is consistent with values reported for other PET radiotracers currently in human use. There was no noticeable bone uptake suggestive of radiotracer defluorination, boding positively for future clinical application studies with [^18^F]LW223. Whole-body human dosimetry results presented here are key for justified large clinical investigations and informed decisions taking into consideration radiation risk.

## Supplementary Information

Below is the link to the electronic supplementary material.

Supplementary Material 1 (DOCX 23.9 KB)

## Data Availability

The datasets generated during and/or analysed during the current study are available from the corresponding author on reasonable request.
