# Supplementary material for: First human whole-body biodistribution and dosimetry analysis of [18F]LW223, a novel TSPO PET radiotracer
Source: Eur J Nucl Med Mol Imaging. 2026 Jan 10;53(6):3951–9. doi: 10.1007/s00259-025-07722-0 (PMC13121222; doi:10.1007/s00259-025-07722-0)
Supplement: Supplementary file 1 — Supplementary Material 1 (DOCX 23.9 KB) [file 259_2025_7722_MOESM1_ESM.docx]

**Supplementary**

**First human whole-body biodistribution and dosimetry analysis of [^18^F]LW223, a novel TSPO PET radiotracer**

Phyo Khaing^1*^, Mark MacAskill^1,2*^, Jianfei Xiao^3^, Shichao Liu^4^, Zhuqin Gu^4^, Xiaohiu Sun^4^, Tao Xu^4^, Norman Koglin^5^, Andrew Stephens^5^, David Newby^1,2^, Yihui Guan^3^, Holly McErlain,^6^ Andrew Sutherland^6^, Gilles D Tamagnan^4^, Fang Xie^3†^, Adriana Alexandre S. Tavares^1,2†^

^1^ Institute for Neuroscience and Cardiovascular Research, University of Edinburgh, Edinburgh, UK

^2^ Edinburgh Imaging, University of Edinburgh, Edinburgh, UK

^3^ Department of Nuclear Medicine and PET Center, Huashan Hospital, Fudan University, Shanghai, China

^4^ XingImaging LLC, New Haven, CT, USA

^5^ Life Molecular Imaging GmbH, Berlin, Germany

^6^ School of Chemistry, University of Glasgow, Glasgow, UK

^*^ Joint first authors; ^†^Joint corresponding authors

**Names and addresses for correspondence:**

| Adriana A. S. Tavares  Institute for Neuroscience and Cardiovascular Research  Queen’s Medical Research Institute  47 Little France Crescent  EH16 4TJ, Edinburgh, UK  **Telephone number:** +44 (0)131 242 6693  **Email:** [adriana.tavares@ed.ac.uk](mailto:adriana.tavares@ed.ac.uk) | Fang Xie  Department of Nuclear Medicine and PET Center  Huashan Hospital  Fudan University  Shanghai, China  **Telephone number:** +86 138 1867 2745  **Email:** [fangxie@fudan.edu.cn](mailto:fangxie@fudan.edu.cn) |
| --- | --- |

**Supplementary Results:**

| **VOI Volume (ccm)** | | | | | | |  |
| --- | --- | --- | --- | --- | --- | --- | --- |
| **Organ** | **Observer 1** | | **Observer 2** | | **Observer 3** | | **%CV** |
|  | **Mean** | **SD** | **Mean** | **SD** | **Mean** | **SD** |  |
| Brain | 1592 | 43 | 1429 | 104 | 1464 | 125 | 5.8 |
| Gallbladder Wall | 63 | 39 | 57 | 34 | 60 | 31 | 5.3 |
| Small Intestine | 3043 | 354 | 1170 | 487 | 2880 | 345 | 43.9 |
| Heart Wall | 273 | 39 | 279 | 49 | 209 | 38 | 15.4 |
| Kidneys | 352 | 69 | 372 | 58 | 348 | 95 | 3.5 |
| Liver | 1440 | 261 | 1216 | 235 | 1760 | 346 | 18.6 |
| Lungs | 1999 | 354 | 2016 | 444 | 2387 | 633 | 10.3 |
| Salivary Glands | 125 | 49 | 97 | 33 | 87 | 40 | 19.2 |
| Red Marrow | 2096 | 242 | 2326 | 350 | 2141 | 310 | 5.6 |
| Spleen | 206 | 33 | 218 | 46 | 217 | 70 | 3.1 |
| Thyroid | 18 | 3 | 15 | 9 | 18 | 8 | 8.8 |
| Urinary Bladder Wall | 234 | 123 | 176 | 101 | 192 | 97 | 15.1 |
|  |  |  |  |  | **Mean %CV** | | 12.9 |

**Supplementary Table 1. Inter-observer VOI volumes and their variance.** %CV= coefficient of variation, n=6.

| **τ values** | | | | | | |  |
| --- | --- | --- | --- | --- | --- | --- | --- |
| **Organ** | **Observer 1** | | **Observer 2** | | **Observer 3** | | **%CV** |
|  | **Mean** | **SD** | **Mean** | **SD** | **Mean** | **SD** |  |
| Brain | 0.035 | 0.006 | 0.031 | 0.008 | 0.032 | 0.007 | 6.8 |
| Gallbladder Wall | 0.023 | 0.028 | 0.022 | 0.027 | 0.025 | 0.030 | 6.1 |
| Small Intestine | 0.201 | 0.045 | 0.101 | 0.061 | 0.189 | 0.064 | 33.3 |
| Heart Wall | 0.023 | 0.005 | 0.023 | 0.005 | 0.018 | 0.003 | 12.1 |
| Kidneys | 0.028 | 0.006 | 0.030 | 0.007 | 0.027 | 0.008 | 4.9 |
| Liver | 0.137 | 0.039 | 0.118 | 0.035 | 0.161 | 0.044 | 15.4 |
| Lungs | 0.058 | 0.012 | 0.056 | 0.015 | 0.068 | 0.017 | 10.2 |
| Salivary Glands | 0.006 | 0.003 | 0.005 | 0.002 | 0.005 | 0.003 | 10.0 |
| Red Marrow | 0.097 | 0.013 | 0.103 | 0.014 | 0.100 | 0.013 | 3.0 |
| Spleen | 0.017 | 0.004 | 0.018 | 0.004 | 0.017 | 0.005 | 3.3 |
| Thyroid | 0.001 | 0.000 | 0.001 | 0.000 | 0.001 | 0.000 | 8.5 |
| Urinary Bladder Wall | 0.028 | 0.015 | 0.025 | 0.014 | 0.027 | 0.016 | 6.2 |
|  |  |  |  |  | **Mean %CV** | | 10.0 |

**Supplementary Table 2. Inter-observer τ values and their variance.** %CV= coefficient of variation, n=6.
